# Mother & Baby Mental Health Inpatient Provision for Wales–Leveraging Mathematical Modelling for More Effective and Responsive Mental Health Services

**DOI:** 10.1192/bjo.2026.11072

**Published:** 2026-06-30

**Authors:** Ollie John, Alka Ahuja, Daniel Gartner, Shane Mills

**Affiliations:** 1 Royal College of Psychiatrists Wales, Cardiff, United Kingdom; 2 Cardiff University, Cardiff, United Kingdom; 3 NHS Wales, Cardiff, United Kingdom

## Abstract

**Aims:**

We evaluated the adequacy of Mother & Baby Unit (MBU) provision for the population of Wales. Primary objectives included assessing historical patterns of patient referrals and admissions, identifying peak demand trends, and projecting future requirements. Additionally, we utilised capacity modelling to evaluate service scenarios and recommend innovations to reduce admission delays.

**Methods:**

We analysed retrospective data on admissions and activity from November 2021 to January 2025. Demand was estimated using epidemiology and live birth statistics from 2023. Travel distances and times were modelled using Google Maps car journey calculations. Furthermore, we in corporated qualitative insights from a Lived Experience Group to define service standards, such as the preferred maximum travel time of one hour.

Working with Cardiff University’s School of Mathematics we developed large language models against scenario analysis.

Our programme has utilised mathematical modelling techniques specifically queuing theory, discrete event simulation (DES), and mathematical programming to ultimately address keychallenges in mental health services – lever aging mathematical modelling for more effective and responsive mental health services.

**Results:**

- Incidence and Admissions: Between 2021 and 2024, 164 mothers from NHS Wales were admitted to MBUs, a figure consistent with clinical projections.
- Service Provision: 81% of admissions were to Uned Gobaith (the only NHS MBU in Wales), which has a 6-bed capacity and is frequently at full occupancy. The remaining 19% of mothers were admitted to units in England.
- Accessibility: While 73% of Welsh mothers travelled less than one hour for treatment, significant regional inequalities exist. Mothers from Betsi Cadwaladr University Health Board travelled the furthest, with an average time of 1 hour 45 minutes.
- Economic Impact: MBU beds are high-cost interventions, with an annual occupancy cost per bed of £350,000–£400,000.

**Conclusion:**

- Current Welsh MBU provision is operating at capacity, often necessitating out-of-area placements.
- To eliminate delays, South Wales requires access to 8–9 beds (up from 6), and North Wales requires 2–3 beds.
- The opening of Seren Lodge in Chester (Autumn 2025) is projected to increase the percentage of North Wales mothers travelling less than one hour from 13% to 69%.
- Future planning must balance inpatient bed expansion with investment in community services to reduce admission needs.
- Mathematical modelling, which has proven effective across various industries, offers a toolbox to help optimising mental health services by improving resource allocation, staff scheduling, and patient care pathways.

